# Food Web Topology in High Mountain Lakes

**DOI:** 10.1371/journal.pone.0143016

**Published:** 2015-11-16

**Authors:** Javier Sánchez-Hernández, Fernando Cobo, Per-Arne Amundsen

**Affiliations:** 1 Department of Arctic and Marine Biology, Faculty of Biosciences, Fisheries and Economics, UiT The Arctic University of Norway, Tromsø, Norway; 2 Department of Zoology and Physical Anthropology, Faculty of Biology, University of Santiago de Compostela, Santiago de Compostela, Spain; 3 Station of Hydrobiology “Encoro do Con”, Castroagudín s/n, 36617 Vilagarcía de Arousa, Pontevedra, Spain; GEOMAR: Helmholtz Center for Ocean Research, GERMANY

## Abstract

Although diversity and limnology of alpine lake systems are well studied, their food web structure and properties have rarely been addressed. Here, the topological food webs of three high mountain lakes in Central Spain were examined. We first addressed the pelagic networks of the lakes, and then we explored how food web topology changed when benthic biota was included to establish complete trophic networks. We conducted a literature search to compare our alpine lacustrine food webs and their structural metrics with those of 18 published lentic webs using a meta-analytic approach. The comparison revealed that the food webs in alpine lakes are relatively simple, in terms of structural network properties (linkage density and connectance), in comparison with lowland lakes, but no great differences were found among pelagic networks. The studied high mountain food webs were dominated by a high proportion of omnivores and species at intermediate trophic levels. Omnivores can exploit resources at multiple trophic levels, and this characteristic might reduce competition among interacting species. Accordingly, the trophic overlap, measured as trophic similarity, was very low in all three systems. Thus, these alpine networks are characterized by many omnivorous consumers with numerous prey species and few consumers with a single or few prey and with low competitive interactions among species. The present study emphasizes the ecological significance of omnivores in high mountain lakes as promoters of network stability and as central players in energy flow pathways via food partitioning and enabling energy mobility among trophic levels.

## Introduction

Ever since the concepts of food chains and food webs were introduced in the in the late 1800s, many studies on feeding relationships have been carried out to address food web complexity and functioning [1]. The importance of omnivorous species as promoters of stability [2–4] has been recognised as a result of their ability to use different food resources leading to reduced inter- and intraspecific competition [4]. In addition, the prevalence of omnivores in food webs may be related to two other important features: firstly, omnivores can have an important impact on energy flows and nutrient cycling [5] and secondly, omnivores can increase the number of links (usually measured as linkage density or the average number of links per species) and hence the food web connectance (i.e., the realized number of links as a proportion of the potential number of links) [3].

Fish are usually at the top of freshwater food chains [6–9]. In lakes, a major pathway of energy transfer is often through pelagic food chains [10], but also benthic prey can be important resources for fish [11,12]. Fish, via their foraging behaviour, are able to modify important food web properties such as for example linkage density, connectance and omnivory [13]. Food web topology in lakes has received attention [14–19], but the majority of the studies have focused on the pelagic zone [15,17–19], and relatively few have included the macroinvertebrates from the littoral and profundal zones [14,16]. The inclusion of the benthic biota in food web analysis allows a broader perspective of ecosystem functioning, especially when it is considered that the pelagic, littoral and profundal zones may be coupled through fish [12,20].

High mountain lakes that are present across the world constitute simple systems due to low species diversity [21–23] and low primary production [24,25]. Originally fish-free alpine lakes have frequently been stocked with salmonid species for recreational purposes, and food webs can be affected by fish introductions because fish are able to modify the structure and composition of both the zooplankton and littoral macroinvertebrate communities [26–28]. To understand the ecosystem functioning of such high mountain lakes, their food web topology has to be explored, but there are still several issues relating to food web structure, prevalence of omnivory and trophic level about which little is known. A notable exception is the study by Harper-Smith *et al*. [16], which demonstrated that the structural complexity of the food web in high elevation lakes in Sierra Nevada (2870–3600 m) is greatest in those that are fish-free. In the present study, we have constructed trophic webs for three high elevation lake systems of Central Spain (lakes Caballeros, Cimera and Grande de Gredos) to include all relevant trophic levels (littoral vegetation, phytoplankton, zooplankton, macroinvertebrates, amphibians and fish). The main objective was to provide, analyse and compare high-resolution topological food webs for the three systems; one lake without fish, one with introduced brook charr *Salvelinus fontinalis* (Mitchill, 1814) (henceforth simply charr) and one with native brown trout *Salmo trutta* Linnaeus, 1758 (henceforth simply trout). More specifically, this study aimed to: (i) quantify important structural network properties of alpine lakes such as food web connectance, number of trophic levels and prevalence of omnivory, (ii) investigate how the structural network properties change when benthic biota are added to complement the pelagic web and complete the lacustrine food web, and (iii) compare the food web topology of these alpine lakes with published information from other lentic systems using a meta-analytic approach. We hypothesised that (i) connectance would be higher in lakes with fish than those without, (ii) structural network properties should be more complex when benthic biota are incorporated, and (iii) food webs in alpine lakes are relatively simple in terms of linkage density and connectance, in comparison with lowland lakes.

## Methods

### Ethics statements

The Servicio Territorial de Medio Ambiente de Ávila (Junta de Castilla y León) granted permission for sampling and protocols were approved by the Institutional Animal Care and Use Committee of the University of Santiago de Compostela (Spain). All procedures conformed to European Union (86/609/EEC) and Spanish (Royal Decree 223/1998) guidelines on animal care and experimentation. All efforts were made to minimize animal stress and suffering during this study. Fish were euthanized by cerebral concussion followed by cervical dislocation to ensure the cessation of life. None of the sampled species were endangered or protected.

### Study area

**Fig 1:**
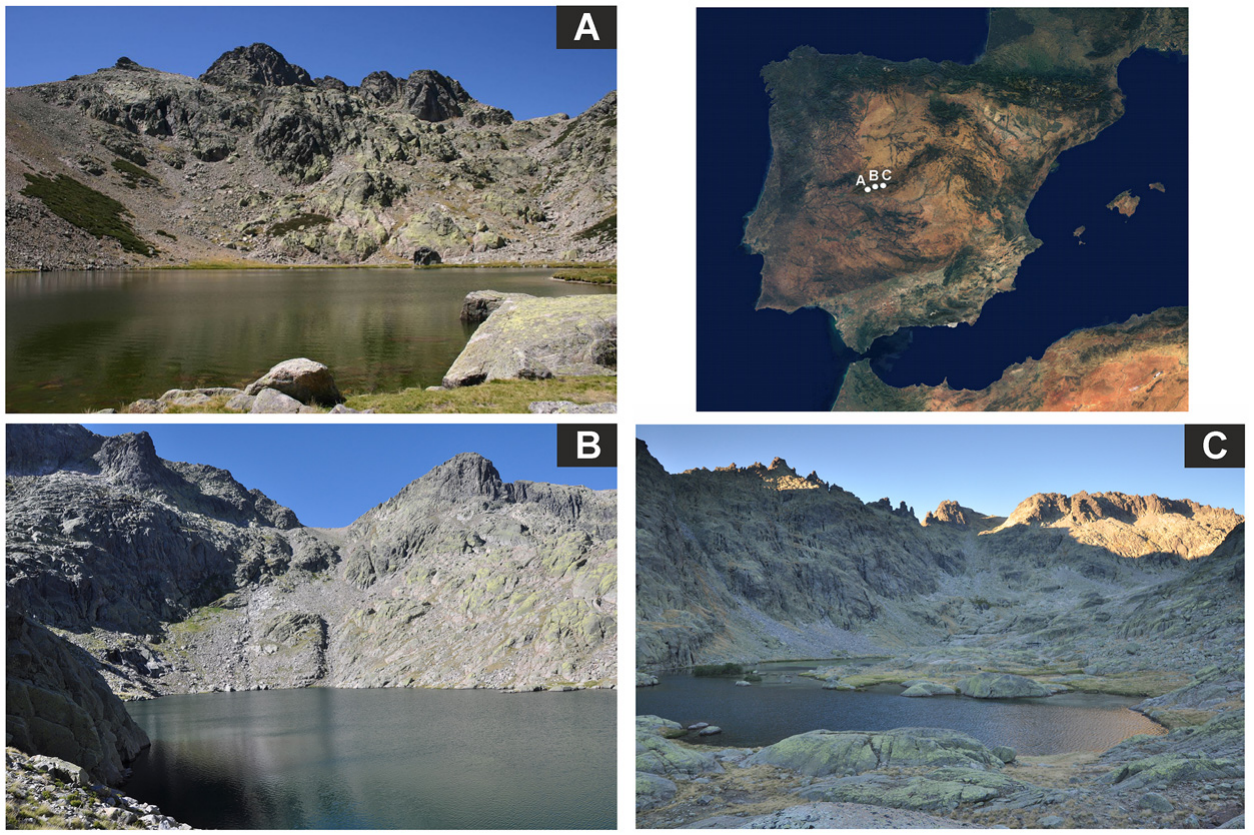
Location of the sampling sites. Location of the sampling sites in the Sierra de Gredos Natural Park (Central Spain). Caballeros (A), Cimera (B) and Grande de Gredos (C) lakes. The upper right part of the figure was taken from a public domain (NASA Earth Observatory, http://earthobservatory.nasa.gov/). The others parts of the figure are owned by JSH.

The study area is located in the Gredos Mountains (Ávila, Central Spain) within the Sierra de Gredos Natural Park, which is part of the Natura 2000 network (Council Directive 92/43/EEC). The topography has origin in tectonic developments during the Alpine Orogeny (Cenozoic), and landscape changes during the Last Glacial Period (Late Pleistocene), when glaciers formed the lakes of the Gredos Mountains [29]. The study involves three high mountain lakes (Caballeros, Cimera and Grande de Gredos) on the northern slope of the Gredos Mountains at altitudes between 1935 and 2140 m.a.s.l. (Fig 1). The lakes are oligotrophic, with conductivities generally below 10 μS/cm [30,31]. The bottom of these lakes is mainly silty with relatively low organic matter content, but in the shoreline area the amounts of sandy bottom and block material are higher [30]. The lakes are ice-covered for 6–7 months from November/December to April/May, with ice cover durations of approximately 185 days for the Grande de Gredos lake and 220 days for Cimera lake [30]. Ice phenology is under the influence of climate change via precipitation and air temperature changes [32]. The three lakes are monomictic with winter inverse stratification during the ice-covered period, whereas after the ice-melt periods, water column mixing predominate [30,31].

Caballeros lake (40°13´N, 5°35´W) with a maximum depth of 5.2 m and a surface area of 14027 m^2^ [30,31], is located in a small granite catchment at 2025 m.a.s.l. [30,31]. The catchment is dominated by *Nardus stricta* grasslands and psicroxerophytic meadows, but with alpine shrub vegetation on the less steep slopes [30,31]. Macrophytes are scarce in the lake, but small areas of *Antinoria agrostidea*, *Isoetes boryana*, *Sparganium angustifolium*, *Callitriche palustris* and *Carex* spp. exist in the littoral zone [33,34]. There are no fish species in the lake.

Cimera lake (40°15´N, 5°18´W) is within a small granite catchment at 2140 m.a.s.l. with exposed rock, i.e. mostly without developed soils but with small *Nardus stricta* grasslands and psicroxerophytic meadows [35]. The lake has a maximum depth of 9.4 m and a surface area of 44900 m^2^ [30,35]. There are no submerged macrophytes, but small areas of water-mosses, mainly *Drepanocladus exannulatus* and *Fontinalis squamosa*, are present in the littoral zone [31]. The levels of polycyclic aromatic hydrocarbons in the superficial sediments of the lake are lower than in lakes in France, Austria, Poland, Slovakia, Ireland and Norway, indicating low contamination by persistent organic pollutants [36]. The only fish present is the charr, which was introduced between 1940 and 1960 and currently has a stable population [35].

Grande de Gredos lake (40°15´N, 5°16´W) is located at 1935 m.a.s.l. The catchment area is mainly rocky with small *Nardus stricta* grassland and psychroxerophytic meadows [37]. The lake has a maximum depth of 6.5 m and a surface area of 63076 m^2^ [31]. The littoral zone is predominantly shallow and there is abundant aquatic vegetation, mainly *Subularia aquatica*, *Isoetes velatum*, *Isoetes boryana*, *Juncus bulbosus*, *Juncus tanageia*, *Callitriche palustris*, *Antinoria agrostidea*, *Ranunculus peltatus*, *Carex nigra*, *Drepanocladus exannulatus*, *Fontinalis squamosa*, *Sphagnum denticulatum* and *Sparganium angustifolium* [30,33,34]. The main anthropogenic impact is from a mountain refuge built in 1971 [37,38]. Native trout are the only fish present in the lake.

### Species compositions

The two primary sources for the data were surveys of macroinvertebrates, amphibians and fish conducted in all three lakes during the summers of 2006 and 2008, and retrieved from publications containing relevant information about the three lakes. Macroinvertebrates were collected from the littoral zone with a pond net by kicking and sweeping (10 samples in each lake), standardised by kicking for 5 minutes. Macroinvertebrates were separated from silt, sand and vegetation and preserved in 96% ethanol for later identification in the laboratory. Species lists were supplemented with information from published studies [30,31,39,40] to establish the final macroinvertebrate data set. Amphibians were monitored by searching for hidden individuals in cavities, soil cracks and under rocks both during daylight and at night (with flashlights). Two anurans (*Bufo spinosus* Daudin, 1803 and *Rana iberica* Boulenger, 1879) and an urodelan, *Salmandra salamandra* (L), were found in the three lakes. Fish were captured by fishing using spinning lures; a total of 49 charr (11–27.2 cm) and ten trout (21–26 cm) were collected. Stomachs were removed and preserved in 96% ethanol for later analysis of stomach contents. Information about phytoplankton and zooplankton compositions were taken from Toro *et al*. [30] and Toro and Granados [31]. The species compositions of each group (phytoplankton, zooplankton, macroinvertebrates, amphibians and fish) in each lake are given as supporting information (S1 File). To study the macroinvertebrate trophic structure, each taxon was assigned to a functional feeding group (*FFG*) based on information from relevant literature [41]. The groups used are based on Merritt and Cummins [42], and species were classified according to Sánchez-Carmona *et al*. [3] as: 1) collectors (feed on fine detritus deposited on the substrate), 2) filterers (feed on fine detritus suspended in the water column), 3) shredders (feed on coarse organic material, such as leaves or wood), 4) predators (feed on other macroinvertebrates) and, 5) scrapers (feed on algae). This study is based on presence-absence data for species in each lake (i.e. the species composition) and does not include considerations of abundance.

### Food web construction

The establishment of feeding links between the species present in the lakes was performed on the basis of our stomach contents analyses (macroinvertebrates and fish) together with information from the literature. Literature searches were conducted using databases (e.g., Web of Science^®^, Dialnet and IngentaConnect) to find information about the feeding habits of the zooplankton, macroinvertebrate, amphibian and fish species present in the lakes. The diets of charr and trout in high mountain lakes are well documented [43–46]. Diet compositions of the amphibian species present in the lakes are also known [47–49]. For the diets of zooplankton [50–52] and macroinvertebrates [41,53,54], studies from a broad geographical range had to be used (see full reference list used in this study for establishing the feeding links between the species in S2 File). Due to a lack of information about whether the phytoplankton species present are unicellular, filamentous, colonial or other forms, most phytoplankton species are considered as being unicellular except for some Chlorophyta (*Pediastrum tetras*, *Scenedesmus* sp., *Dictyosphaerium* sp., *Dictyosphaerium enrenbergianum*, *Dictyosphaerium subsolitarium*, *Hyalotheca dissiliens*, *Sphaerocystis schroeteri* and *Spirogyra* sp.), some Bacillariophyceae (*Aulacoseira distans*, *Asterionella formosa*, *Navicula cryptocephala*, *Navicula* sp., *Fragilaria crotonensis* and *Tabellaria flocculosa*), some Cyanophyta (*Anabaena* sp., *Pseudanabaena* sp., *Lyngbya* sp. and *Bicosoeca alaskana*) and some Chrysophyceae (*Dinobryon bavaricum*, *Dinobryon sertularia*, *Salpingoeca* sp. and *Synura* sp.) that are mainly filamentous or colonial species [55,56]. We assumed that filamentous and colonial forms of phytoplankton species cannot be utilized as food by zooplankton because of their size [57], whereas zooplankton could graze on unicellular species. Rotifers have only been recorded in Caballeros lake (*Keratella quadrata* and *Conochilus* sp.) [31]. The trophic base in the benthic habitats were aggregated into the following groups: coarse organic material (leaves, wood), fine detritus, macrophytes, water-moss and periphyton. Fungi or bacteria are able to break down coarse organic material and fine detritus [58], but microorganisms have not been included in this study.

The topological food webs of the three lakes were established according to Amundsen *et al*. [18], consisting of an *n x n* matrix on *n* species, with predators as columns and prey as rows, and with binary entries (0 or 1) in the matrix indicating whether a predator eats a prey species. Then, food web images were produced with FoodWeb3D software developed by the Pacific Ecoinformatics and Computational Ecology Lab [59,60] and available at http://www.foodwebs.org/. The trophic link matrices used for food web construction are given as supporting information (S1–S3 Datasets). To characterise food web topology, a suite of 23 properties was calculated (Table 1). We calculated the number of trophic levels, linkage density and connectance using previously described methods [18,19]. We paid attention to generalization (*GenSD*, “average number of prey eaten per predator in the web”) and vulnerability (*VulSD*, “average number of predators per prey in the web”) [61], that are widely-reported [3,62,63]. When *GenSD* is higher than *VulSD*, it means that there are few consumers with numerous prey and many consumers with a single prey [3]. In line with other studies, trophic similarity (*Sim*) was calculated to address the trophic overlap of food webs [3,63]. The measurement of trophic overlap using a similarity index between every pair of taxa in a food web was proposed by Martinez [14]; index values vary from zero (when two taxa have no common predators or common prey) to one (when two taxa have the same set of predators and prey) [14]. Williams and Martinez [64] recommended the combination of traditional food web properties with analyses of mean short-weighted trophic level (*MeanSWTL*) and mean shortest chain to a basal species (*MeanShortChn*), because these parameters can provide valuable information about energy flow. *MeanSWTL* can indicate how many steps energy must take to get from an energy source to a consumer [1] and *MeanSWTL* has been used to calculate the trophic level averaged across taxa in food webs [62,63]. To calculate *MeanSWTL*, the FoodWeb3D software assigns a value for basal taxa as one, obligate herbivores have trophic level two, and higher level consumers have a value based on their feeding relationships to other levels; more detail about how this index is calculated can be found in previous works [63,64]. Topological food webs were established separately for the pelagic zone and the complete system (pelagic and benthic biota), which allowed us to compare network complexity between the pelagic and complete networks. In addition, topological food webs for Cimera and Grande de Gredos lakes were also constructed without fish species in order to make their structure compatible and comparable with Caballeros lake (fishless lake). This approach allowed us to examine whether fish predation could modify the structure of the food webs in high mountain lakes.

**Table 1:** Food web properties.

Metric	Definition
Richness (*S*)	Taxonomic richness of the food web
Lower trophic taxa (*S*_*l*_)	Fraction of species that are lower trophic taxa (terrestrial organic material, fine detritus, periphyton, macrophytes and water-moss)
Phytoplankton richness (*S*_*p*_)	Fraction of species that are phytoplankton
Zooplankton richness (*S*_*z*_)	Fraction of species that are zooplankton
Macroinvertebrate richness (*S*_*m*_)	Fraction of species that are macroinvertebrate
Amphibian richness (*S*_*a*_)	Fraction of species that are amphibian
Fishes richness (*S*_*f*_)	Fraction of species that are fishes
Links (*L*)	Number of links between individuals
Linkage density (*D*)	*D = L/S*
The potential number of links (*Lp*)	*Lp = S* ^*2*^
Directed connectance (*C*)	*C = L/S* ^*2*^
Top species (*T*)	Fraction of species that have no predators
Intermediate species (*I*)	Fraction of species that have both predators and prey
Basal species (*B*)	Fraction of species that are not consumers
Omnivory (*Omn*)	Fraction of species that are omnivores (consume prey from more than one trophic level)
Herbivores (*Herb*)	Fraction of species that are herbivores (only consume basal species)
Cannibalism (*Can*)	Fraction of species that are cannibals
*MeanSWTL*	Mean short-weighted trophic level [64]
*MeanShortChn*	Mean shortest chain to a basal species
*GenSD*	Generality standard deviation (standard deviation of the number of resources per species)
*VulSD*	Vulnerability standard deviation (standard deviation of the number of consumers per species)
Trophic similarity (*Sim*)	Mean Jaccardian similarity [14]
*MaxSim*	Maximum Jaccardian similarity [14]

Definition of the 23 food web properties calculated for the three high mountain lakes.

### Meta-analytic approach

Meta-analysis refers to a set of methods used to analyse or compare results from different studies [65]. The data from the three lakes were compared to food web statistics gathered from 18 published food webs from lentic systems [14–19]. The meta-analysis was carried out separately for complete food webs [14,16], and pelagic food webs [15,17–19], and was implemented by using common food web properties presented in all the 18 published food webs: species richness (*S*), number of links (*L*), linkage density (*D*) and connectance (*C*). The data used are provided as supporting information (S1 Table). A principal component analysis (*PCA*) was used to explore differences in the food web properties among systems. In addition, data matrices were analysed using between-class analysis in order to explore the affinity of the systems in the *PCA*. Between-class analysis is a method used to enhance the understanding of similarities between grouped classes [66,67]. The data analysis involved in three steps: (i) the *dudi*.*pca* function was employed to perform a principal component analysis of a data frame, (ii) the *scatter* function was used for plotting the food web properties axes, the position of lakes and the eigenvalues of the *PCA*, and (iii) the *s*.*class* function was used to test for differences in the projection of the food web properties with ellipses and gravity centers grouped by study. In order to explore the statistical significance of the between-group analysis, a permutation test (Monte-Carlo test) was used [68]; the test was considered statistically significant at *P* < 0.05. Graphical outputs and permutation analysis were computed with the ADE4 library implemented in R freeware [69]. The ADE4 library [70] is at http://cran.es.r-project.org/.

## Results

### Food web topology

**Fig 2:**
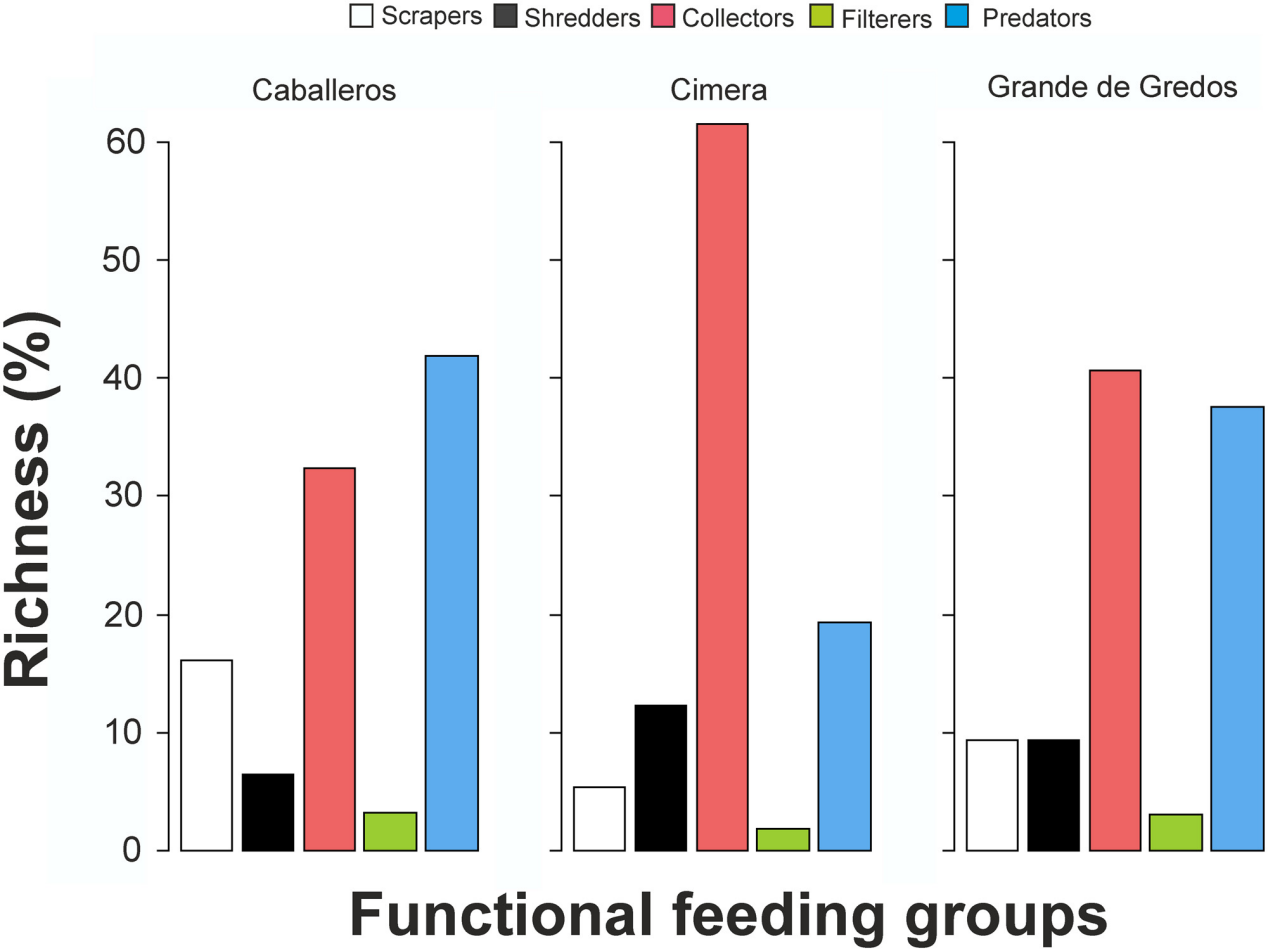
Functional feeding groups. Functional feeding groups of the macroinvertebrate community in each lake.

The combination of benthic biota with the pelagic network to give the complete food web increased the number of links (by 4.9 and 14.3 times in Caballeros and Cimera, respectively) and resulted in an increase in food-web characteristics such as linkage density and *GenSD* (Table 2). In addition, when fishless topologies were constructed for the three lakes, *GenSD* and *VulSD* values were lower in the fishless condition, whereas there were no marked differences in connectance (Table 2). The remainder of this section refers to the complete networks, but information about the pelagic and fishless food-web metrics can be obtained from Table 2. Taxonomic richness of the food webs varied from 64 to 96 (Table 2), of which the majority were macroinvertebrates (48% in Caballeros and 67% in Cimera) and phytoplankton (41% in Grande de Gredos). In all three lakes, Chironimidae dominated the taxonomic richness of the macroinvertebrate community with 19.4%, 40.6% and 56.1% of the total number of taxa in the respective lakes (Caballeros, Grande de Gredos and Cimera). Collectors and predators were proportionally the most abundant functional feeding groups, while filter feeders were scarce and were only represented by *Pisidium casertanum* (Poli, 1791) (Fig 2).

**Table 2:** Food web topology.

	Caballeros		Cimera			Grande de Gredos		
	Pelagic	Complete	Pelagic	Fishless	Complete	Pelagic	Fishless	Complete
Richness (*S*)	21	64	17	84	85	43	95	96
Lower trophic taxa (*S*_*l*_)*	0	0.13	0	0.06	0.06	0	0.17	0.17
Phytoplankton richness (*S*_*p*_)	0.76	0.27	0.59	0.15	0.15	0.86	0.41	0.41
Zooplankton richness (*S*_*z*_)	0.24	0.08	0.35	0.07	0.07	0.12	0.05	0.05
Macroinvertebrate richness (*S*_*m*_)	0	0.48	0	0.68	0.67	0	0.34	0.33
Amphibian richness (*S*_*a*_)	0	0.05	0	0.04	0.04	0	0.03	0.03
Fishes richness (*S*_*f*_)	0	0	0.06	0	0.01	0.02	0	0.01
Links (*L*)	70	344	45	580	645	101	498	534
Linkage density (*D*)	3.3	5.4	2.6	6.9	7.6	2.3	5.2	5.6
The potential number of links (*Lp*)	441	4096	289	7056	7225	1849	9025	9216
Directed connectance (*C*)	0.16	0.08	0.16	0.08	0.09	0.05	0.06	0.06
Top species (*T*)	0	0.09	0.06	0.06	0.01	0.02	0.06	0.02
Intermediate species (*I*)	0.24	0.57	0.24	0.77	0.82	0.07	0.42	0.46
Basal species (*B*)	0.76	0.34	0.75	0.17	0.16	0.91	0.52	0.52
Herbivores (*Herb*)	0	0.18	0	0.08	0.06	0	0.10	0.05
Omnivory (*Omn*)	0.64	0.84	0.64	0.90	0.91	0.72	0.90	0.93
Cannibalism (*Can*)	0.09	0.09	0.12	0.06	0.06	0.02	0.04	0.04
*MeanSWTL*	2.60	2.71	3.58	2.76	2.89	3.37	2.94	3.17
*MeanShortChn*	2.43	2.25	2.64	2.23	2.15	2.88	2.44	2.51
*GenSD*	0.53	0.65	0.53	0.93	0.84	0.39	0.76	0.73
*VulSD*	1.88	1.13	1.76	1.78	1.79	3.61	1.52	1.52
Trophic similarity (*Sim*)	0.43	0.14	0.37	0.25	0.26	0.59	0.19	0.19
*MaxSim*	1	1	1	1	1	1	1	1

Values of structural properties food webs for the three high mountain lakes. See Table 1 for definitions of each property.

*Lower trophic taxa includes organic material, detritus, periphyton and littoral vegetation.

**Fig 3:**
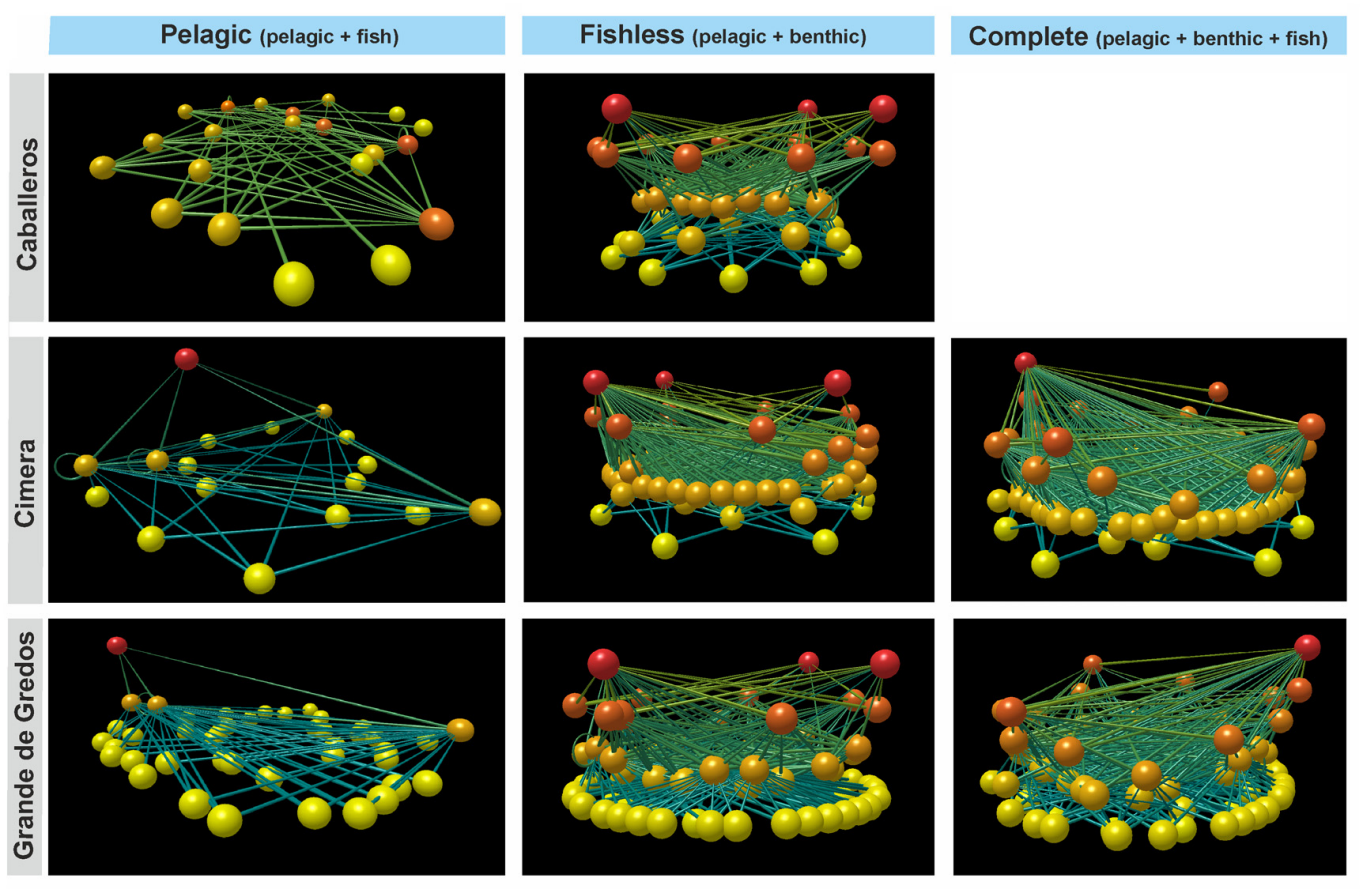
Alpine food webs. Three-dimensional visualization of the complexity of food webs from the three studied lakes in Central Spain. Image produced with software Foodweb3D available from the Pacific Ecoinformatics and Computational Ecology Lab (http://www.foodwebs.org/), and written by RJ Williams [59,60]. Balls are nodes that represent species and sticks are the links that connect balls through consumption. The vertical axis corresponds to trophic level, basal trophic levels (yellow) are on the bottom; upper trophic levels are on the top (red).

Structural network properties (number of links, connectance and linkage density) were always higher in Cimera (charr-lake) than in the other two lakes (Table 2). The food webs had from 4096 to 9216 potential links, of which only 5.8–8.9% were observed. In all lakes, the food webs included a large proportion of species that are both predators and prey, i.e. that are intermediate species (>45%, Table 2). The proportion of basal species was high in Grande de Gredos (52%), but much lower in the other two lakes (16% and 34% in Cimera and Caballeros, respectively), and the percentage of top species was higher in fishless Caballeros (9%) than in the two lakes where fish are present (2% and 1% in Grande de Gredos and Cimera, respectively). The species at the highest trophic levels in the food webs were amphibians in Caballeros, and charr and trout in Cimera and Grande de Gredos, respectively (red balls in the Fig 3). Six trophic levels were identified in lakes with fish species; basal level (terrestrial organic material, fine detritus, periphyton, macrophytes and water-moss), phytoplankton, zooplankton, macroinvertebrates, amphibians and fish. Five trophic levels were recognised in the fishless lake (Caballeros). Interestingly the studied high mountain food webs were characterised by high proportions of omnivores regardless of lake type (Table 2), and the prevalence of omnivores was always above 80%. The fraction of species that were cannibals was low, and never above 0.10 (Table 2).

Measures of trophic level characteristics (*MeanSWTL* and *MeanShortChn*) were similar, but there were some differences (Table 2). The lakes with fish (Grande de Gredos and Cimera) had the highest *MeanSWTL*-values, and Cimera tended to have shorter food chains (lowest *MeanShortChn*-value) than the other two lakes (Table 2). The average number of prey species eaten per predator species (*GenSD*) and the mean number of predators per prey (*VulSD*) were highest in Cimera and lowest in Caballeros (Table 2). Values of *VulSD* were higher than those for *GenSD* in all the food webs. Some taxa such as colonial phytoplankton, nematodes and *Gordius* sp. were not consumed by predators. With regard to trophic similarity (*Sim*), numerical values were closer to 0 than to 1 in all three lakes (values between 0.14 to 0.26), meaning that many taxa did not have common predators or common prey. The maximum trophic similarity (*MaxSim*) was the same in the three lakes (Table 2).

### Meta-analytic approach

**Fig 4:**
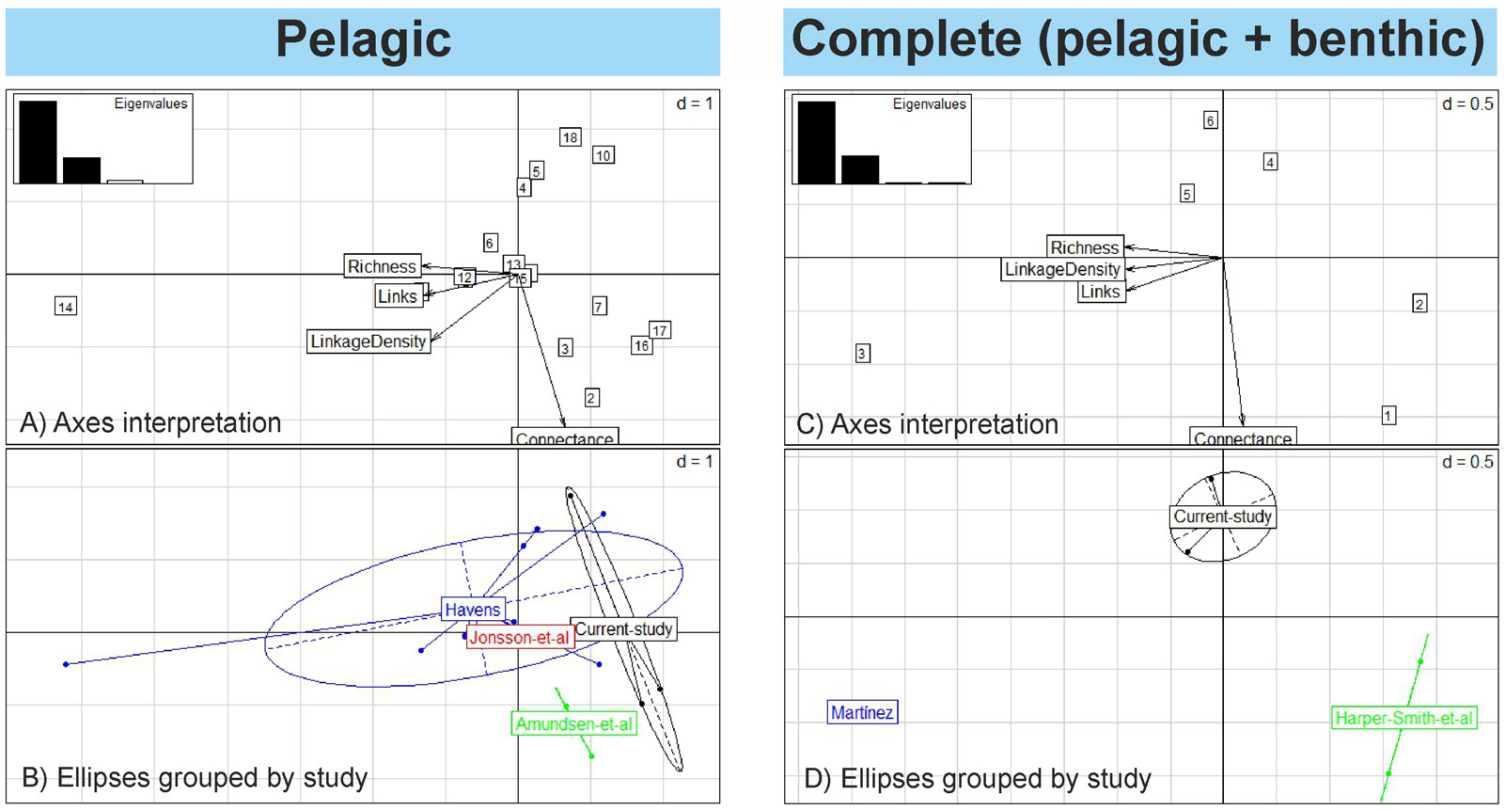
Meta-analytic approach. Principal component analysis (*PCA*) plot based on four food web properties. This is a composed plot, made of: A and C parts- a plot of food web properties axes, position of lakes and eigenvalues projected into *PCA*, B and D parts- the projection of the food webs with ellipses and gravity center grouped by study.

Food web properties of the networks addressed in the present study were compared to published data for 18 food webs from lentic systems (Fig 4). The first two axes of the *PCA* explained 97.8 and 99% of the total variability of the pelagic and complete food webs, respectively (Table 3). The interpretation of the axes in the *PCA*, i.e. position of lakes, and position of food web properties and eigenvalues, are shown in the upper parts of (Fig 4A and 4C). The permutation test confirmed that the difference among groups was significant among the complete food webs (Fig 4D, *P* = 0.004), but not among the pelagic food webs (Fig 4B, *P* = 0.168). Thus, the comparison among pelagic food webs shows that our networks were similar to those in other systems. For the complete food webs, our webs differ from those recorded for other systems (Fig 4D). The estimated food web properties (i.e., richness, number of links, linkage density and connectance) are low in comparison with values reported by Martinez [14]; but in contrast, the richness, number of links between individuals and linkage density were higher than values reported by Harper-Smith *et al*. [16] (values are shown in S1 Table).

**Table 3:** Scores of eigenvalues.

	Model 1 (pelagic zone)	Model 2 (pelagic and benthic zones)
Eigenvalue of axis 1	2.974	2.954
Eigenvalue of axis 2	0.936	1.007
Eigenvalue of axis 3	0.087	0.032
Eigenvalue of axis 4	0.003	0.008

Scores of eigenvalues extracted by principal component analysis for each model. Two models are shown; firstly including literature from the pelagic zone (model 1) and secondly including literature using both pelagic and benthic zones (model 2).

## Discussion

### Food web topology

Our study confirms that the food web topology of alpine lakes may be relatively simple in terms of structural network properties like linkage density and connectance in comparison with lowland lakes, but our findings also demonstrate that the studied mountain lakes clearly were dominated by a high proportion of omnivores and species at intermediate trophic levels. Food webs often have between four and six trophic levels [18,19,71]. In our case, six trophic levels were revealed in two out of the three lakes addressed in the study, whereas five trophic levels were identified in fishless Caballeros. *MeanSWTL* was lower in the fishless system than in the lakes with fish. This could be related to the high predation capacity of fish as generalistic and opportunistic foragers, here represented by trout and charr, that are able to feed on a large variety of taxa and increase the number of feeding links in a food web [8,44,72,73]. Our *MeanSWTL*-values were relatively high compared to those previously reported for lakes, streams, estuaries, marine habitats and terrestrial systems [3,62]. For example, Dunne *et al*. [62] reported *MeanSWTL*-values for food webs in lakes in the range 2–2.7, but our *MeanSWTL*-values were higher than this. For the Iberian Peninsula, the only comparable information is from food webs in streams [3], and our *MeanSWTL*-values were higher than for the lotic webs. These differences could relate to the high proportion of omnivores and species at intermediate trophic levels identified in our study.

The food webs in our high mountain lakes were characterized by a high proportion of omnivores and intermediate trophic level species, suggesting that these taxa may play an important role in ecosystem functioning of alpine lakes. In general, omnivory is common in food webs [64], and high levels of omnivory have been observed in temperate lakes [74]. Thompson *et al*. [75] reviewed the prevalence of omnivory in different ecosystems (streams, lakes, terrestrial and marine systems), and found that lakes in general have intermediate prevalence of omnivory relative to marine systems (higher) and streams (lower). The present study demonstrates that omnivory in alpine lakes can be very important with values always above 80% in the complete networks. Our values were higher than those for Mediterranean shallow-oligohaline lakes [76], subtropical lakes [77], Mediterranean streams [3], North American zooplankton food webs of lakes [78] and some marine food webs from USA and UK [64], but similar to some temperate lakes [74] and marine food webs [62,79]. Several researchers have claimed that a high prevalence of omnivorous species in a food web likely has ecological significance because a high predation capacity increases linkage density and promotes the stability and persistence of webs [3,76,80,81]. Omnivorous species exploit resources at multiple trophic levels, and this might be important to reduce inter- and intraspecific competition, enhance the partitioning of food and promote the mobility of energy transfer. Taking the opposite point of view, omnivory could also increase competition by increasing the potential for species to share prey [82,83]. However, it seems more reasonable to posit that omnivory reduces competition for food in the studied lakes; a conclusion supported by the trophic similarity index, which revealed that trophic overlap was low in all three lakes. The high prevalence of intermediate and omnivorous species could reflect an important role in energy flow in high mountain food webs with them acting as important vectors for transferring energy inputs from both allochthonous and autochthonous sources into the lake food webs.

Contrary to our expectations, the food web complexity in the Caballeros network (fishless system), measured as directed connectance, was similar to Cimera lake (charr system), but higher than Grande de Gredos lake (trout system). Food web complexity in lakes has previously been related to the presence or absence of fish species [16,84]. Harper-Smith *et al*. [16] pointed out that the connectance in high mountain lakes with fish was 24% lower than in fishless systems. On the other hand, Parker and Huryn [84] have shown that fish are able to increase food chain length and network complexity. Recent studies have also demonstrated that when fish were introduced into freshwater ecosystems, they are able to alter the topology of food webs, increasing linkage density, connectivity and complexity [19,85]. Thus, it is reasonable to posit that food webs might differ between fishless lakes and lakes with fish. In our case, some differences were found in the number of links and linkage density among networks (always Cimera > Grande de Gredos > Caballeros), but when the food topology was constructed without the fish species, linkage density was higher in Caballeros than in Grande de Gredos (Cimera > Caballeros > Grande de Gredos). These findings differ somewhat from the higher linkage density found in fishless systems in North-American alpine lakes [16]. Therefore, our study highlights that fish predation may modify the structure of the food web. Trout is a native species in Grande de Gredos and charr was introduced in Cimera, and our results indicate that the top-down effects may be higher in alpine lake systems with trout as the top predator than those in which charr have been introduced. However, this conclusion should be treated with caution, and more studies would be needed to corroborate or refute this.

When fish are present, vulnerability and generality of food webs tend to be higher. Our results are consistent with Schoener [61]’s conclusion that standard deviations for generalization distributions (*GenSD*) are lower than those for vulnerability distributions (*VulSD*). This conclusion was arrived by following the study of terrestrial, marine benthic, marine pelagic, marine estuarine, intermediate estuarine, and lotic and lentic freshwater systems. By contrast, some researchers have found that *GenSD* values exceeded *VulSD* values in high mountain lake [16] and stream food webs [3]. It has been considered that when this occurs (i.e., *GenSD*>*VulSD*), it means that there are few consumers with numerous prey and many consumers with a single prey [3]. Our study revealed the opposite, as in all three lakes there were many consumers with numerous prey and few consumers with a single prey (Fig 3). Also, comparing our *GenSD* and *VulSD* values with published data from alpine lakes, our *GenSD* and *VulSD* values were lower and higher, respectively, than reported by Harper-Smith *et al*. [16]. Trophic similarity (*Sim*) gives us valuable information about whether or not taxa in food webs have common predators and prey [14]. In our case, the mean value of this metric was lowest in the fishless lake, but was low (below 0.30) for all three lakes, indicating that trophic overlap between taxa tended to be low in all three studied lakes.

Although we have gained some knowledge about high-altitude lake systems, several ecological aspects are still left unexplored. In particular, limited information is available about the topology of alpine lacustrine food webs, including both the pelagic and benthic compartments [16]. Our study aimed to assist in filling this knowledge gap and enhance the understanding of alpine ecosystems. It is noteworthy that the results of our study highlight the important role of omnivores for the functioning of high mountain lake food webs. How projected changes in climate will influence such food-web structure is not certain, but food chain lengths of modern lakes seem to be shorter than in ancient lakes as a consequence of the prevalence of omnivores and herbivores [86]. Increases in water temperature are likely to lead to increases in eutrophication and stratification with a reduction of oxygen in the hypolimnion; such changes may result in the extirpation of salmonids [87]. Therefore, knowledge about food web topology and functioning in high mountain lakes is required in order to allow predictions to be made about how energy flow and food-web interactions could be affected by ongoing climate change.

### Meta-analytic approach

The outcome of the meta-analysis indicated that the studied lakes had structural food web properties that differed from several North American lakes. The complete food webs in the high mountain lakes were simple compared to those in Little Rock Lake, a mesotrophic and low altitude lake in northern Wisconsin [14], but more complex than those of high mountain lakes in central Sierra Nevada, which had lower richness, linkage density and number of feeding links between species [15]. On the other hand, the multivariate *PCA* demonstrated that our pelagic networks were similar to other pelagic systems [15,17–19]. Although our meta-analysis revealed some similarities and dissimilarities in structural network properties between pelagic and complete food webs, the results of the analyses should be taken with some caution because they are focused around a small number of topology metrics based on the number of links and species richness. Future studies might extend the present work to investigate homogeneity among food webs, including observations from more alpine lakes and more metrics. Our findings revealed that when the biota and trophic interactions in the benthic zone are combined with the pelagic food web to provide the complete food web for a lake system, the network properties change. It should, however, be kept in mind that the topology of these alpine lakes was constructed by combining observational data and published literature. Consequently, some of the feeding links between species might not have taken place in the studied lakes. Thus, by including theoretical links from a number of different studies, the number of links and thereby other food web properties (e.g., connectance, linkage density, omnivory, etc) might be overestimated, but our topologies could be used as an initial reference for temperate high-mountain lakes.

## Supporting Information

S1 Dataset Data links of Caballeros food web. Refer to S1 File for food web naming conventions. (TXT)

S2 Dataset Data links of Cimera food web. Refer to S1 File for food web naming conventions. (TXT)

S3 Dataset Data links of Grande de Gredos food web. Refer to S1 File for food web naming conventions. (TXT)

S1 File Data nodes of complete food webs, including list of species and basic information about each nodes. (XLS)

S2 File Full reference list used in this study for the food web construction. (DOCX)

S1 Table Food web properties used for the comparative study. Richness (*S*), links (*L*), linkage density (*D*) and connectance (*C*). When the reference includes several values for the same lake, food web properties are shown as average values with the range in parenthesis. (DOCX)
