# Correction: Food Web Topology in High Mountain Lakes

**DOI:** 10.1371/journal.pone.0145214

**Published:** 2015-12-11

**Authors:** The PLOS ONE Staff

## Notice of Republication

S1 File, S2 File, and S1 Table were published in error. The errors were corrected in the HTML version of this article on December 1, 2015, and the corrected files are available in the supporting information of this article. The publisher apologizes for the errors.

## Supporting Information

S1 Correct Corrected S1 File, S2 File, and S1 Table. (ZIP)
