# Supplementary material for: Food Web Topology in High Mountain Lakes
Source: PLoS One. 2015 Nov 16;10(11):e0143016. doi: 10.1371/journal.pone.0143016 (PMC4646624; doi:10.1371/journal.pone.0143016)
Supplement: S2 File — (DOCX) [file pone.0143016.s005.docx]

S2 File. Full reference list used in this study for the food web construction.

Adrian, R. & Frostm T.M. 1992. Comparative feeding ecology of *Tropocyclops prasinus mexicanus* (Copepoda, Cyclopoida). J. Plankton Res. 14: 1369–1382.

Anderson, N.H. 1976. Carnivory by an aquatic detritivore, *Clistoronia magnifica* (Trichoptera : Limnephilidae). Ecology 57: 1081–1985.

Aussel, J.-P. & Linley, J.R. 1994. Natural food and feeding behavior of *Culicoides furens* larvae (Diptera: Ceratopogonidae). J. Med. Entomol. 31: 99–104.

Balseiro, E.G., Modenutti, B.E. & Queimaliños, C.P. 2001. Feeding of *Boeckella gracilipes* (Copepoda, Calanoida) on ciliates and phytoflagellates in an ultraoligotrophic Andean lake. J. Plankton Res. 23: 849–857.

Barrabin, J.M. 2000. The Rotifers of Spanish reservoirs: Ecological, systematical and zoogeographical remarks. Limnetica 19: 91–114.

Bogatova, I.B. 1951. Quantitative data on the feeding of *Cyclops strenuus* Fischer and *Cyclops viridis* Jurine. Inst. ozer. rech. ryb. Khoz. 1: 163–76.

Bowers, J. A. 1980. Feeding habits of *Diaptomus ashlandi* and *Diaptomus sicilis* in Lake Michigan. Int. Revue ges. Hydrobiol. Hydrogr. 65: 259–267.

Bowker, D.W.; Warehorn, M.T. & Learner, M.A. 1983. The selection and ingestion of epilithic algae by *Nais elinguis* (Oligochaeta: Naididae). Hydrobiologia 98: 171–178.

Bradley, M.D.K. & Reynolds, J.D. 1987. Diet of the leeches Erpobdella octoculata (L) and Helobdella stagnalis (L) in a lotic habitat subject to organic pollution. Freshwat. Biol. 18: 267–275.

Braverman, Y. 1994. Nematocera (Ceratopogonicae, Psychodidae, Simuliidae and Culicidae) and control methods. Rev. Sci. Tech. Oie. 13: 1175–1199.

Brock, M.L., Wiegert, R.G. & Brock, T.D. 1969. Feeding by *Paracoenia* and *Ephydra* (Diptera: Ephydridae) on the Microorganisms of Hot Springs. Ecology 50: 192–200.

Brown, D.S. 1961. The food of the larvae of *Chloeon dipterum* L. and *Baetis Rhodani* (Pictet) (Insecta. Ephemeroptera). J. Anim. Ecol. 30: 55–75.

Callisto, M., Gonçalves, J.F. & Graça, M.A.S. 2007. Leaf litter as a possible food source for chironomids (Diptera) in Brazilian and Portuguese headwater streams. Rev. Bras. Zool. 24: 442–448.

Calow, P. 1973. Food of *Ancylus fluviatilis* (Mull), a littoral stone-dwelling, herbivore. Oeeologia 13: 113–133.

Campeny, R. & Montori, A. 1995. Feeding of an Iberian population of *Bufo bufo* during the reproductive period. In: Llorente, G.A., Montori, A., Santos, X., Carretero, M. A. (Eds.). *Scientia Herpetologica: papers submitted from the 7th Ordinary General Meeting of Societas Europaea Herpetologica, Barcelona, September* 15-19, 1993. Asociación Herpetológica Española, Barcelona. pp. 172-175.

Cavalli, L., Chappaz, R. & Gilles, A. 1998. Diet of Arctic charr (*Salvelinus alpinus* (L.)) and Brown trout (*Salmo trutta* L.) in sympatry in two high altitude alpine lakes. Hydrobiologia 86: 9–17.

Cavalli, L., Chappaz, R. Bouchard, P. & Brun, G. 1997. Food availability and growth of the brook trout, *Salvelinus fontinalis* (Mitchill), in a French Alpine lake. Fisheries. Manag. Ecol. 4: 167–177.

Chang, K.-H., Doi H., Nishibe, Y. & Nakano, S.-I. 2010. Feeding habits of omnivorous Asplanchna: comparison of diet composition among *Asplanchna herricki*, *A. priodonta* and *A. girodi* in pond ecosystems. J. Limnol. 69: 209–216.

Clitherow, L.R., Carrivick, J.L. & Brown, L.E. 2013. Food Web Structure in a Harsh Glacier-Fed River. PLoS ONE 8: e60899

Cummins, K.W., Merritt, R.W. & Andrade, P.C.N. 2005. The use of invertebrate functional groups to characterize ecosystem attributes in selected streams and rivers in south Brazil. Stud. Neotrop. Fauna and Environ. 40: 69–89.

Debenham, M.L. 1989. Family Ceratopogonidae, In: Evenhuis, N.L. (ed.), Catalog of the Diptera of the Australasian and Oceanian regions. Bishop Museum Press, Honolulu & E.J. Brill, Leiden. pp. 226-251.

Descy, J.-P., Frost, T.M. & Hurley. J.P. 1999. Assessment of grazing by the freshwater copepod *Diaptomus minut*es using carotenoid pigments: A caution. J. Plankton Res. 21: 127–145.

Díaz-Paniagua, C. 1987. Estudio en cautividad de la actividad alimenticia de las larvas de siete especies de anuros. Rev. Esp. Herpetol. 2: 189–197.

Díaz-Paniagua, C. 1989. Larval diets of two anuran species, *Pelodytes punctatus* and *Bufo bufo*, in SW Spain. Amphibia-Reptilia 10: 71–75.

Elliott, J.M. & Humpesch, U.H. 2010. Mayfly Larvae (Ephemeroptera) of Britain and Ireland: Keys and a Review of their Ecology. Freshwater Biological Association Scientific Publications 66: 152 pp.

Elliott, J.M. 1996. British Freshwater Megaloptera and Neuroptera. A key with ecological notes*.* Freshwater Biological Association, Scientific Publication Nº. 54. 68 pp.

Elliott, J.M. 2006. Critical periods in the life cycle and the effects of a severe spate vary markedly between four species of elmid beetles in a small stream. Freshwat. Biol. 51: 1527–1542.

Elliott, J.M. 2008. The ecology of Riffle Beetles (Coleoptera: Elmidae). Freshwater Reviews 1:189–203.

Eloranta, A.P., Knudsen R., & Amundsen, P.-A. 2013. Niche segregation of coexisting Arctic charr (*Salvelinus alpinus*) and brown trout (*Salmo trutta*) constrains food web coupling in subarctic lakes. Freshwat. Biol. 58: 207–221.

Fuentes, N., Güde, H. & Straile, D. 2013, Importance of allochthonous matter for profundal macrozoobenthic communities in a deep oligotrophic lake. Int. Rev. Hydrobiol. 98: 1–13.

Galizzi, M.C., Zilli F. & Marchese, M. (2012). Diet and functional feeding groups of Chironomidae (Diptera) in the Middle Paraná River floodplain (Argentina). Iheringia 102: 117–121.

García-Chicote J., Rojo, C. & Rodrigo, M.A. 2007. Alimentación de *Acanthocyclops robustus:* Un caso de canibalismo. Limnetica 26: 265–276. (In Spanish with abstract in English)

Gibbs, K.E. & Mingo, T.M. 1986. The life history, nymphal growth rates, and feeding habits of *Siphlonisca aerodromia* Needham (Epherneroptera: Siphlonuridae) in Maine. Can. J. Zool. 64: 427–430.

Giller, P.S. 1986. The natural diet of the Notonectidae: field trials using electrophoresis. Ecol. Entomol. 11: 163–172

Guerrero, F., Perez-Mellado, V., Gil, M.J. & Lizana, M. 1990. Food habits and trophic availability in the high mountain population of the spotted salamander from Spain (*Salamandra salamandra almanzoris*) (Caudata: Salamandridae). Fol. Zool. 89: 841–853.

Guiry, M.D. & Guiry, G.M. 2014. AlgaeBase. World-wide electronic publication, National University of Ireland, Galway. http://www.algaebase.org; last accessed date on 11 November 2014.

Gulati, R.D., Bronkhorst, M. & Van Donk, E. 2001. Feeding in *Daphnia galeata* on *Oscillatoria limnetica* and on detritus derived from it. J. Plankton Res. 23: 705–718*.*

Ha, J.-Y. & Hanazato, T. 2009. Role of interference from *Daphnia* and predation by cyclopoid copepods in zooplankton community structure: experimental analysis using mesocosms. Plankton Benthos Res. 4: 147–153.

Henriques-Oliveira, A.L., Nessimian, J.L. & Dorvillé, L.F.M. 2003. Feeding habits of chironomid larvae (Insecta: Diptera) from a stream in the Floresta da Tijuca, Rio de Janeiro, Brazil. Braz. J. Biol. 63: 269–281.

Hildrew, A.G., Townsend C.R. & Hasham, A. 1985. The predatory Chironomidae of an iron-rich stream: feeding ecology and food web structure. Ecol. Entomol. 10: 403–413.

Jackson, R.R. & Walls, E.I. 1998. Predatory and scavenging behaviour of Microvelia macgregori (Hemiptera: Veliidae), a water‐surface bug from New Zealand. New Zeal. J. Zool. 25: 23–28.

Jannot, J.E., Wissinger, S.A. & Lucas. J.R. 2008. Diet and a developmental time constraint alter life-history trade-offs in a caddis fly (Trichoptera: Limnephilidae). Biol. J. Linn. Soc. 95: 495–504.

Johansson A. & Nilsson A.N. 1992. *Dytiscus Zatissimus* and *D. circumcinctus* (Coleoptera, Dytiscidae) larvae as predators on three case-making caddis larvae. Hydrobiologia 248: 201–213.

Jones, J.R.E. 1950. A further ecological study of the River Rheidol The food of the common insects of the main-stream. J. Anim. Ecol. 159–174.

Kampe, H., König-Rinke, M., Petzoldt, T. & Benndorf, J. 2007. Direct effects of *Daphnia*-grazing, not infochemicals, mediate a shift towards large inedible colonies of the gelatinous green alga *Sphaerocystis schroeteri*. Limnologica 37: 137–145.

Knisely K. & Geller W. 1986. Selective feeding of four zooplankton species on natural lake phytoplankton. Oecologia 69: 86–94.

Kosnicki, E. & Burian, S. 2003. Life history aspects of the mayfly *Siphlonurus typicus* (Ephemeroptera: Siphlonuridae) with a new application for measuring nymphal development and growth. Hydrobiologia 510: 131–146.

Lacasse, S. & Magnan, P. 1992. Biotic and abiotic determinants of the diet of the brook trout, *Salvelinus fontinalis*, in lakes of the Laurentian Shield. Can. J. Fish. Aquat. Sci. 49: 1001–1009.

Lizana, M. 1990. Ecología de *Bufo bufo* en la Sierra de Gredos. Ph.D. Thesis, Universidad de Salamanca, Salamanca.

Lizana, M., Ciudad, M.J. & Pérez-Mellado, V. 1986. Uso de los recursos tróficos en una comunidad ibérica de anfibios. Rev. Esp. Herpetol. 1: 207–271.

López-Rodríguez, M.J. & Tierno de Figueroa, J.M. 2006. Estudio de la dieta otoñal de la ninfa de *Protonemura meyeri* (Pictet, 1842) (Plecoptera, Nemouridae) en Río Blanco (Granada, España). Acta Granatense 4/5: 41–44. (In Spanish with abstract in English)

López-Rodríguez, M.J., Figueroa, J.M. & Alba-Tercedor, J. 2010. Comparative Study of the Nymphal Biology of Two Coexisting Species of Mayflies (Insecta: Ephemeroptera) in a Mediterranean Stream in Southern Europe. Int. Rev. Hydrobiol. 95: 58–71.

Majka, C.G. & Kenner, R.D. 2009. The Gyrinidae (Coleoptera) of the Maritime Provinces of Canada: new records, distribution, and faunal composition. In: Majka CG, Klimaszewski J (Eds) Biodiversity, Biosystematics, and Ecology of Canadian Coleoptera II. ZooKeys 22: 355–372.

Martínez, G. 2000. Conducta alimentaria de *Daphnia ambigua* Scourfield 1947, *Moina micrura* Kurz 1874 y *Ceriodaphnia dubia* Richard 1895 (Cladocera) frente a un gradiente de concentración de alimento. Rev. Chil. Hist. Nat. 73: 47–54.

Massaro, F.C, Negreiros, N.F. & Rocha, O.A. 2013. search for predators and food selectivity of two native species of *Hydra* (Cnidaria: Hydrozoa) from Brazil. Biota Neotrop. 13: 35–40.

Mathis, W.N. & Simpson, K.W. 1981. Studies of Ephydrinae (Diptera: Ephydridae), V: The genera Cirrula Cresson and Dimecoenia Cresson in North America. Smithsonian Contrib. Zool. 329: 1–51.

McElhone, MJ. 1980. Some factors influencing the diet of coexisting, benthic, algal grazing Naididae Oligochaeta. Can. J. Zool. 584: 481–487.

Mihuci, T. & Toetz, D. 1994. Determination of diets of alpine aquatic insects using stable isotope*s* and gut analysis. Am. Midl. Nat. 131: 146–155.

Moore, J.W. 1978. Importance of algae in the diet of the oligochaetes *Lumbriculus variegatus* Muller and *Rhyacodrilus sodalis* Eisen. Oecologia 35: 357–363.

Murdoch, W.W., Scott, M. & Ebsworth, P. 1984. Effects of the general predator Notonecta (Hemiptera) upon a freshwater community. J. Anim. Ecol. 53: 791–808.

Murillo, J. & Recasens, L. 1986. Hábitos alimentarios de *Sigara lateralis* (Heteroptera, Corixidae). Misc. Zool. 10: 135–140. (In Spanish with abstract in English)

Oganjan, K., Virro, T. & Lauringson, V. 2013. Food spectrum of the omnivorous rotifer Asplanchna priodonta in two large northeastern European lakes of different trophy. Oceanol. Hydrobiol. St. 42: 314–323.

Ohba, S. 2009a. Ontogenetic dietary shift in larvae of *Cybister japonicus* (Coleoptera: Dytiscidae) in Japanese rice fields. Environ. Entomol. 38: 856–860.

Ohba, S. 2009b. Feeding habits of the diving beetle larvae *Cybister brevis* *Aube* (Coleoptera: Dytiscidae) in Japanese wetlands. Appl. Entomol. Zool. 44: 447–453.

Ohba, S., Trang Huynh, T.T., Kawada, H., Loan Luu, L., Tran Ngoc, H., Le Hoang, S., Higa, Y. & Takagi, M. 2011. Heteropteran insects as mosquito predators in water jars in southern Vietnam. J Vector Ecol. 36: 170–174.

Olejniczak, I., Boniecki, P., Jablonski, P. & Sang-don, L. 2007. Diet of water striders (*Gerris lacustris* L. 1758) in a rice field near Seoul, Korea. J. Asia Pac. Entomol. 10: 85–88.

**Oscoz**, J., **Galicia**, D. & **Miranda**, R. (Eds.) 2011. Identification Guide of Freshwater Macroinvertebrates of Spain. Springer Dordrecht Heidelberg London New York.

Palmer C.G. & O’Keeffe J.H. 1992. Feeding patterns of four macroinvertebrate taxa from the headwaters of the Buffalo River, Eastern Cape. Hydrobiologia 228: 157–173.

Polegatto, C.M. & Froehlich, C.G. 2001 Functional morphology of the feeding apparatus of the nymph of *Farrodes* sp. (Ephemeroptera: Leptophlebiidae). Acta Zool. 82: 165–176.

Pope, R.J., Gordan, A.M. & Kaushik, N.K. 1999. Leaf litter colonization by invertebrates in the littoral zone of a small oligotrophic lake. Hydrobiologia 392: 99–112.

Ranta, E. & Espo, J. 1989. Predation by the rack-pool insects *Aretoeorisa carinata, Calicorixa produeta* (Het. Corixidae) and *Potamaneetes griseostriatus* (Col. Dytiscidae). Ann. Zool. Fennici 26: 53–60.

Reilly, P. & McCarthy, T.K. 1990. Observations on the natural diet of Cymatia bonsdorfi (C. Sahlb.) (Heteroptera: Corixidae): an immunological analysis. Hydrobiologia 196: 159–166.

Reinhold, J.O., Hendriks, A.J., Slager, L.K. & Ohm, M. 1999. Transfer of microcontaminants from sediment to chironomids and the risk for the pond bat *Myotis dasycneme* (Chiroptera) preying on them. Aquat. Ecol. 33: 363–376.

Reiso, R. & Brittain, L.E. 2000. Life cycle, diet and habitat of *Polvcentropus flavomaculatus, Plectrocnemia conspersa* and *Rhyacophila nubila* (Trichoptera) in Øvre Heimdalen, Jotunheimen Mountains, Norway. Norw. J. Entomol. 47: 113–124.

Rosa, G.M., Laurentino, T. & Madeira, M. 2012. Field observation of foraging behavior by a group of adult diving beetles *Agabus* (*Gaurodytes*) *bipustulatus* preying on an adult *Lissotriton boscai*. Entomol. Sci. 15: 343–345.

Sánchez-Hernández, J. & Amundsen, P.-A. 2015. Trophic ecology of brown trout (*Salmo trutta* L.) in subarctic lakes. Ecol. Freshwat. Fish 24: 148–161.

Sánchez-Hernández, J. & Cobo, F. 2011. Summer food resource partitioning between four sympatric fish species in Central Spain (River Tormes). Fol. Zool. 60: 189–202.

Sánchez-Hernández, J. & Cobo, F. 2008. Importancia y calidad nutritiva de las presas terrestres en la alimentación del salvelino, *Salvelinus fontinalis* (Mitchill, 1914). Cuadernos Abulenses 37: 389–410. (In Spanish with abstract in English)

Sánchez-Hernández, J. & Cobo, F. 2012. Summer differences in behavioural feeding habits and use of feeding habitat among brown trout (Pisces) age classes in a temperate area. Ital. J. Zool. 79: 468–478.

Sánchez-Hernández, J., Cobo, F. & González, M.A. 2007. Biología y la alimentación del salvelino, *Salvelinus fontinalis* (Mitchill, 1914), en cinco lagunas glaciares de la sierra de Gredos (Ávila, España). NACC 16: 129–144. (In Spanish with abstract in English)

Sanseverino, A.M. & Nessimian, J.L. 2008. The food of larval Chironomidae (Insecta, Diptera) in submerged litter in a forest stream of the Atlantic Forest (Rio de Janeiro, Brazil). Acta Limnol. Bras. 20:15–20.

Schmid-Araya, J.M. & Schmid, P.E. 1995. Preliminary results on diet of stream invertebrate species: the meiofaunal assemblages. Jber. Biol. Stn Lunz 15: 23–31.

Sephton, T.W. 1987. Some Observations on the Food of Larvae of *Procladius bellus*. (Diptera: Chironomidae). Aquat. Insects 9: 195–202.

St Clair RM 1994. Diets of some larval Leptoceridae (Trichoptera) in south-eastern Australia. Aust. J. Mar. Freshw. Res. 45: 1023–1032.

Sutcliffe, D.W. 1962. The composition of haemolymph in aquatic insects. J. Exp. Biol. 39: 325–344.

Sutton, M.F. 1951. On the food, feeding mechanism and alimentary canal of Corixidae (Hemiptera, Heteroptera). Proc. Zool. Soc. Lond. 121: 465–499.

Tachet, H., Richoux, P., Bournaud, M. & Usseglio-Polatera, P. 2002. Invertebratébrés d’eau douce. Systematique, biologie, écologie. CNRS Editions. Paris.

Tate, A.W. & Hershey, A.E. 2003. Selective feeding by larval dytiscids (Coleoptera: Dytiscidae) and effects of fish predation on upper littoral zone macroinvertebrate communities of arctic lakes. Hydrobiologia 497:13–23.

Tavares, A.F. & Williams, D.D. 1990. Life histories, diet, and niche overlap of three sympatric species of Elmidae Coleoptera in a temperate stream. Can. Entomol. 122: 563–577.

Tavares-Cromar, A.F. & Williams, D.D. 1997. Dietary overlap and coexistence of chironomid larvae in a detritus-based stream. Hydrobiologia 354: 67–81.

Townsend, C.R. & Hildrew, A.G. 1977. Predation strategy and resource utilisation by Plectrocnemia conspersa (Curtis) (Trichoptera: Polycentropodidae). Proceedings of the 2nd International Symposium on Trichoptera, pp. 283–290. Junk, The Hague.

Townsend, C.R. & Hildrew, A.G. 1979. Resource partitioning by two freshwater invertebrate predators with contrasting foraging strategies. J. Anim. Ecol. 48: 909–920.

Travers, S.E. 1993. Group foraging facilitates food finding in a semi-aquatic hemipteran, *Microvelia austrina* Bueno (Hemiptera: Veliidae). Pan-Pac. Entomol. 69: 117–121.

Van den Bosch, F. & Gabriel, W. 1991. The impact of cannibalism on the population dynamics of cyclopoid copepods. Verh. Internat. Verein Limnol. 24: 2848–2850.

Villalobos, M.J. & González, E.J. 2006. Estudios sobre la biología y ecología de *Ceriodaphnia cornuta* Sars: una revisión. Interciencia 31: 351–357. (In Spanish with abstract in English)

Ward, A.F. & Williams, D.D. 1986. Longitudinal zonation and food of larval chironomids (Insecta: Diptera) along the course of a river in temperate Canada. Ecography 9: 48–57.

Wehr, J.D. & Sheath, R.G. 2003. Freshwater Algae of North America, Ecology and Classification. Academic Press, San Diego (USA).

Wellnitz, T.A. & Ward, J.V. 1998. Does light intensity modify the effect mayfly grazers have on periphyton?. Freshwat. Biol. 39: 135–149.

Yan, Y. & Li, X. 2007. Production dynamics and life cycle of dominant chironomids (Diptera, Chironomidae) in a subtropical stream in China: adaptation to variable flow conditions in summer and autumn. Chin. J. Oceanol. Limnol. 25: 330–342.

Young, J.O. 1980. A serological investigation of the diet of Helobdella stagnalis (Hirudinea: Glossiphoniidae) in British lakes. J. Zool. 192: 467–488.

Young, J.O., Martin, A.J. & Seaby, R.M.H. 1993. Competitive interactions between the lake-dwelling leeches *Glossiphonia complanata* and *Helobdella stagnalis*: an experimental investigation of the significance of a food refuge. Oecologia 93: 156–161.

Zamora–Muñoz, C., Tierno de Figueroa, T. & Alba-Torcedor, J. 1999. Factores relacionados con la distribución de *Sialis nigripes* Pictet, 1865 Megaloptera, Sialidae) en la cuenca alta del rio Genil (sur de España). Zool. Baetica 10: 193–202.
