# Supplementary material for: Food Web Topology in High Mountain Lakes
Source: PLoS One. 2015 Nov 16;10(11):e0143016. doi: 10.1371/journal.pone.0143016 (PMC4646624; doi:10.1371/journal.pone.0143016)
Supplement: S1 Table — (DOCX) [file pone.0143016.s006.docx]

S1 Table. Complete list of references and food web properties used for the comparative study. Richness (*S*), links (*L*), linkage density (*D*) and connectance (*C*). When the reference includes several values for the same lake, food web properties are shown as average values with the range in parenthesis.

| Lake | Reference (study) | Country | Richness (*S*) | Links (*L*) | Linkage density *(D*) | Connectance (*C*) |
| --- | --- | --- | --- | --- | --- | --- |
| Takvatn | Amundsen et al. (2009) | Norway | 37 | 198 | 5.36 | 0.145 |
| Takvatn | Amundsen et al. (2013) | Norway | 34 (31-37) | 181.5(165-198) | 5.3 (5.32-5.35) | 0.159 (0.172-0.145) |
| Sierra Nevada | Harper-Smith et al. (2005) | North America | 24 (20-28) | 75.2 (43-108) | 3.0 (2.1-3.8) | 0.17 (0.15-0.2) |
| Alford | Havens (1992) | North America | 56 | 219 | 3.9 | 0.070 |
| Balsam | Havens (1992) | North America | 53 | 182 | 3.4 | 0.065 |
| Beaver | Havens (1992) | North America | 61 | 327 | 5.4 | 0.088 |
| Big Hope | Havens (1992) | North America | 30 | 120 | 4.0 | 0.133 |
| Bridge Brook | Havens (1992) | North America | 75 | 552 | 7.4 | 0.098 |
| Chub Pond | Havens (1992) | North America | 65 | 417 | 6.4 | 0.099 |
| Connery | Havens (1992) | North America | 30 | 60 | 2.0 | 0.067 |
| Hoel | Havens (1992) | North America | 49 | 254 | 5.2 | 0.106 |
| Long | Havens (1992) | North America | 65 | 416 | 6.4 | 0.098 |
| Stink | Havens (1992) | North America | 53 | 280 | 5.3 | 0.100 |
| Little | Havens (1992) | North America | 176 | 2009 | 11.4 | 0.065 |
| Tuesday | Jonsson *et al*. (2005) | North America | 50 | 268 | 5.4 | 0.107 |
| Little Rock | Martínez (1991) | North America | 182 | 2366 | 13.0 | 0.144 |
